# Constitutive nitric oxide synthases deficiency impairs cyclobutane pyrimidine dimer repair following solar UV exposure in cells and mice

**DOI:** 10.1111/php.70024

**Published:** 2025-08-31

**Authors:** Veronica Bahamondes Lorca, Yuxi Zhou, Christina Athans, Hailey Payne, Madison Wright, Zeinab Feyyaz, Lingying Tong, Dawn L. Sammons, Shiyong Wu

**Affiliations:** ^1^ Department of Chemistry and Biochemistry Ohio University Athens Ohio USA; ^2^ Departamento de Tecnología Médica, Facultad de Medicina Universidad de Chile Santiago Chile; ^3^ Honor Tutorial College Ohio University Athens Ohio USA; ^4^ Oakview Dermatology Athens Ohio USA; ^5^ Present address: Moffitt Cancer Center Tampa FL 33612 USA; ^6^ Present address: Nationwide Children's Hospital Columbus OH 43205 USA

**Keywords:** constitutive nitric oxide synthase (cNOS), DNA damage, DNA repair, photocarcinogenesis, solar ultraviolet radiation (sUV)

## Abstract

Solar ultraviolet (sUV) radiation is a major environmental factor that induces DNA damage, promoting skin aging and carcinogenesis. The formation of cyclobutane pyrimidine dimers (CPDs) is one of the most prevalent forms of UV‐induced DNA lesions, playing a central role in skin photocarcinogenesis. Constitutive nitric oxide synthase (cNOS), responsible for basal nitric oxide (NO^˙^) production, has been implicated in various cellular processes, including the DNA damage response. However, the role of cNOS in modulating DNA repair post‐UV exposure has not been explored. In this study, we investigated the impact of cNOS deficiency on CPD repair following sUV exposure using both in vivo and in vitro models. SKH‐1 hairless wild‐type and nNOS^+/−^/eNOS^−/−^ (cNOS‐deficient) mice were chronically exposed to sUV, revealing significantly exacerbated skin lesions in cNOS‐deficient animals. Primary fibroblasts and skin explants derived from these mice, as well as HEK293 cells with stable cNOS overexpression, were analyzed for CPD formation and repair dynamics. Our findings show that cNOS knockout leads to impaired CPD repair, with CPD levels persisting longer in cNOS‐deficient cells and tissues compared with wild‐type controls. Reintroduction of cNOS expression in HEK293 cells accelerated CPD clearance early post‐sUV exposure, suggesting a protective role for cNOS in the DNA repair process. These results highlight cNOS as a critical modulator of UV‐induced DNA damage repair and underscore its potential role in mitigating skin carcinogenesis.

## Graphical abstract

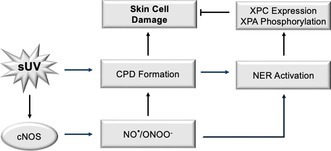

**Abbreviations**
6‐4PPs: pyrimidine (6‐4) pyrimidone photoproducts
cNOS: constitutive nitric oxide synthase
CPDs: cyclobutane pyrimidine dimers
DDR: DNA damage response
eNOS: endothelial NOS
NER: Nucleotide Excision Repair
nNOS: neuronal NOS
NO˙: nitric oxide
SCC: squamous cell carcinoma
sUV: Solar ultraviolet

## INTRODUCTION

Solar ultraviolet (UV) radiation is a major environmental stressor that causes a wide range of detrimental effects in the skin, such as aging and carcinogenesis. The damage induced to the DNA by both UVA and UVB radiations is mainly due to the formation of pyrimidine dimers and oxidative‐derived lesions.^1^, ^2^ The pyrimidine dimers induced most abundantly post‐UV exposure corresponding to the cyclobutane pyrimidine dimers (CPDs) and the pyrimidine (6‐4) pyrimidone photoproducts (6‐4PPs), both of which have a significant role in the carcinogenesis process.^1^, ^2^, ^3^

The cellular response to UV‐induced DNA damage involves a complex network of repair mechanisms, with critical roles played by various enzymes and proteins. The Nucleotide Excision Repair (NER) pathway is activated when DNA damage is induced by ultraviolet radiation exposure and is the primary mechanism of DNA damage repair involved in the removal of CPDs and6‐4 PPs.^4^, ^5^

Upon UV exposure, constitutive nitric oxide synthase (cNOS), including endothelial NOS (eNOS) and neuronal NOS (nNOS), is rapidly activated.^6^, ^7^ These enzymes are involved in the production of nitric oxide (NO^˙^), a signaling molecule that plays a key role in various cellular processes, including DNA repair. Studies have highlighted the critical role of NO^˙^ in regulating DNA repair pathways following UV damage.^8^, ^9^, ^10^ Excessive NO^˙^ levels can cause DNA damage and disrupt DNA repair mechanisms by modifying DNA and proteins involved in DNA repair.^11^, ^12^, ^13^, ^14^ Conversely, we hypothesize that insufficient NO^˙^ levels may also impair the DNA damage response (DDR), based on evidence that NO^˙^ can either activate or inhibit ATR signaling in a cell‐type‐specific context.^15^ This suggests a potential bidirectional role for NO^˙^ in DDR regulation, emphasizing the importance of maintaining a proper balance of NO^˙^ for efficient DNA repair.

This report explores the role of cNOS in skin carcinogenesis and DNA repair post‐solar ultraviolet (sUV) exposure. To investigate the role of cNOS in UV‐induced skin damage and CPD repair, we employed various experimental models, including SKH‐1 mice, primary mouse fibroblasts, mouse skin explants, and HEK293 cells. These models allowed us to assess the impact of cNOS genetic deficiency on skin lesions and CPD formation and repair following solar UV exposure. By comparing wild‐type and cNOS knockout mice, fibroblasts isolated from the skin of these mice, as well as cNOS‐null HEK293 cells and their stable cNOS‐expressing counterparts, we aimed to elucidate the involvement of cNOS in the photocarcinogenic response and DNA repair mechanisms after UV radiation.

## MATERIALS AND METHODS

### sUV irradiation

For irradiation, skin samples were exposed to a single (explant analysis) or multiple (in vivo analysis) doses of 180 mJ/cm^2^ of solar UVB, while a single dose of 200 mJ/cm^2^ was used for cell culture studies. Solar UV (sUV) irradiation was performed using a previously characterized UVA/UVB solar lamp (UVA‐340, Q‐Lab Corporation).^16^ Briefly, the lamp emits radiation from ~280 nm, with peak intensity at 340–345 nm. Its spectrum closely simulates natural sunlight, with ≤10% UVB (280–315 nm) and ≥90% UVA (315–400 nm).^16^ After the lamp warmed up, UVB irradiance was measured using a UVX‐31 sensor (280–340 nm, UVP, Inc.), with a correction factor applied to account for UVA contribution.^16^ Skin explants and cell culture samples were irradiated for approximately 20 min at a dose rate of 0.16 ± 0.01 mW/cm^2^. Mice were irradiated for approximately 37 min at a dose rate of 0.08 ± 0.01 mW/cm^2^. During irradiation, cell culture samples were maintained in 1X PBS to prevent desiccation. Minimal cell death was observed under these conditions at the time of experimentation.

### SKH‐1 mice model

The cNOS KO SKH1 mice were developed using CRISPR/Cas9 technology. Briefly, heterozygous cNOS (nNOS^+/−^/eNOS^+/−^) SKH‐1 mice (purchased from TransViragen Inc. #Ex328, Research Triangle Park, NC, USA) were generated at the UNC Animal Model Core Facility. Due to the fact that the genes encoding nNOS and eNOS are 46 centimorgan (cM) apart on Chromosome 5, double knockout was generated by knocking out two genes simultaneously using the CRISPR/Cas system. Heterozygous mice were then bred in‐house to generate the other desired genotypes. The animals were housed in groups (2–4/cage) and undisturbed (except for normal cage cleaning) with food and water available ad libitum except during experimental procedures. Adult animals (~8 weeks old) were used to start sUV irradiation. All efforts were made to minimize animal suffering. All protocols were approved and are in accordance with international guidelines for animal experimentation.

### Isolation of primary fibroblast

After the mice either died naturally or were euthanized in accordance with IACUC guidelines, skin samples were collected from wild‐type (WT) and nNOS^−/−^/eNOS^−/−^ (DKO) SKH‐1 mice that had not been subjected to any experimental treatments or UV irradiation. The collection protocol was adapted from Walmsley et al.^17^ The mice were first sterilized in 70% ethanol for at least 15 min. The dorsal skin was then carefully dissected, avoiding the limbs and any large fat deposits typically found in the abdominal and lateral abdominal regions. The skin was cut into approximately 1.5 cm^2^ pieces and rinsed with a solution of 1X PBS and Gentamicin (50 ng/mL, Gibco, 15750‐060). Next, the skin samples were treated with cold 0.5% dispase solution (5 U/mL dispase in Hanks' Balanced Salt Solution, #07913, Stemcell™) in DMEM medium (Corning™, Cell Gro™, 10‐013‐CV) and incubated at 37°C for 45 min. After rinsing with PBS, the skin layers were separated, and the epidermis was discarded. The dermis was then incubated in a 15 mL tube with 10 mL of collagenase type IV (1 mg/mL, Gibco, 17104‐019) in DMEM to isolate individual cells. After centrifuging and collecting the pellet, the cells were seeded and cultured in DMEM medium (Corning™, Cell Gro™, 10‐013‐CV) supplemented with 10% v/v FBS and 1% v/v penicillin/streptomycin at 37°C with 5% CO_2_. To prevent microbial and fungal contamination, Primocyn™ (100 μg/mL, Invivogen, #ant‐pm‐05) and Fungin™ (10 μg/mL, Invivogen, ant‐fn‐1) were added to the cell culture medium for 5 days.

### Skin explants collection, maintenance, and sUV irradiation

Skin samples were obtained from wild‐type (WT) and nNOS^+/−^/eNOS^−/−^ SKH‐1 mice that had not been subjected to any experimental treatments or UV irradiation. Tissues were collected post‐euthanasia from animals sacrificed for unrelated, IACUC‐approved procedures, following the method outlined by Payne et al.^18^ Briefly, after sterilizing the mice with 70% ethanol, the skin was removed from the dorsal region. The fat and connective tissue were carefully separated from the skin and placed onto a synthetic polycaprolactone scaffold.^19^ For irradiation, the skin was exposed to a single dose of sUV, using 180 mJ/cm^2^ of UVB from a UVA/UVB solar lamp (UVA‐340, Q‐Lab Corporation).^16^ After irradiation, the samples were incubated at 37°C with 5% CO_2_ for the specified time periods (20, 40, and 60 min). During incubation, the skin‐scaffold pieces were placed in culture medium with the dermis facing downward and the dry epidermis facing outward, ensuring proper orientation during the experiment. One skin explant per time point for each genotype was assayed, fixed in 10% paraformaldehyde, and embedded in paraffin for subsequent processing and CPD detection.

### SKH‐1 mice irradiation and skin lesion quantification

SKH‐1 wild‐type and nNOS^+/−^ eNOS^−/−^ (cNOS knockout) mice were irradiated with sUV at 180 mJ/cm^2^ of UVB from a UVA/UVB solar lamp (UVA‐340, Q‐Lab Corporation),^16^ three times a week for 28 weeks before being euthanized 6 h after the last sUV irradiation treatment. During irradiation, mice were moved to a dedicated irradiation chamber and exposed in small groups within open containers to ensure uniform sUV exposure. The containers have no lids or obstructions, and no food or water devices were present during irradiation to eliminate shade or hiding opportunities. Mice were not anesthetized, and sessions lasted approximately 37 min per exposure, based on a dose rate of ~0.08 ± 0.01 mW/cm^2^. Mice were monitored continuously throughout the procedure. All the procedures followed IACUC protocols. After euthanasia, dorsal skin tissue was collected and immediately stored at −80 °C for subsequent protein analysis. For skin lesion quantification, the percentage of area with lesion in a selected region of interest (ROI) was calculated using ImageJ. In this analysis, mice were randomized and blinded during lesion assessment. Images were taken immediately after euthanasia at week 28. For the analysis, the sample sizes for each group are as follows: female WT = 9 control and 11 sUV, male WT = 12 control and 12 sUV, female nNOS^+/−^/eNOS^−/−^ = 4 control and 5 sUV, male nNOS^+/−^/eNOS^−/−^ = 9 control and 7 sUV.

### PCR for genotype characterization

To verify the genotype of the mice and fibroblasts, PCRs (polymerase chain reaction) were performed using DNA extracted from the tails of the mice, as well as from the fibroblasts. Briefly, DNA extraction solutions 1 (25 mM NaOH, 2 mM EDTA) and 2 (40 mM Tris–HCl) were freshly prepared. 100 μL of solution 1 was added to the cell pellet or tail clipping and heated at 95°C for 1 h. Then, 100 μL of solution 2 was added, and the samples were vortexed, centrifuged, and the supernatant containing DNA was collected for PCR. DNA (15 μg/μL) was used with GoTaq® Green Master Mix (M712, Promega).

PCR was performed using an Eppendorf 5331 MasterCycler Gradient Thermocycler under the following conditions: initial denaturation at 95°C for 2 min; 14 cycles of 95°C for 30 s, 72°C for 30 s (reducing 1°C per cycle), and 72°C for 30 s; followed by 24 cycles of 95°C for 30 s, 58°C for 30 s, and 72°C for 30 s; with a final extension at 72°C for 5 min. PCR products were analyzed on a 4% agarose gel using 1X TBE buffer. For the characterization, the following primers were used:

**nNOS KO:** Fw: 5′‐ACTCTCAGAGTGAAAGACACGCTAGG‐3′ and Rv: 5′‐ACTTGCCGTTAGGACATTTGCTAA‐3′,

**nNOS WT:** Fw: 5′‐ACTCTCAGAGTGAAAGACACGCTAGG‐3′ and Rv: 5′‐CCAACAAGATCAGCACTGTTATTTGTG‐3′,

**eNOS KO:** Fw: 5′‐ACCTGATCCTGGCCTTTGTGTC‐3′ and Rv: 5′‐GGGAGAAGGTGATTTGTGACAGG‐3′,

**eNOS WT:** Fw: 5′‐ACCTGATCCTGGCCTTTGTGTC‐3′ and Rv: 5′‐CAGGAGCTTCCTGGATCTGTTCA‐3′.

### Overexpression of cNOS and cell culture of HEK293 cells

Human HEK293 (ATCC, CRL‐1573™) and HEK293 overexpressing cNOS (eNOS and nNOS stable expressing cell line) were grown in Eagle's Minimal Essential Medium (ATCC, 30‐2003™) supplemented with 10% v/v fetal bovine serum and 1% v/v penicillin/streptomycin at 37°C with 5% CO_2_. Briefly, for constructing the HEK293 stable cell line overexpressing cNOS (cNOS OE), HEK293 cells (cNOS null) were co‐transfected with pReceiver‐M02‐nNOS (GeneCopoeia) and pcDNA3‐eNOS‐GFP (Addgene # 22444)^20^ using Lipofectamine 2000 and selected with G418 for two weeks. The colonies were collected, and the expression level of nNOS and eNOS was determined by western blot.

### DNA extraction and ELISA

DNA extraction was performed using the kit DNeasy Blood and Tissue Kit (Qiagen, 69,504), following the manufacturer's indications. The formation of CPDs in the DNA samples was detected and quantified using the OxiSelect™ UV‐Induced DNA Damage ELISA Kit (Cell Biolabs Inc., STA‐322), following the manufacturer's protocol. For CPD detection, standard curves were generated using serial dilutions of known concentrations of CPD‐DNA standard (Cell Biolabs Inc., Part No. 232203), and sample concentrations were calculated based on these curves. To ensure the samples fall into the range of the standard curve, 0.8 μg/mL of DNA from fibroblasts was used, while 1.9 μg/mL of DNA from HEK293 cells was used. Four experimental replicates were analyzed, with each DNA sample assayed in duplicate. Absorbance was read at 450 nm using a plate reader BioTek Cytation 5.

### Immunostaining of cyclobutane pyrimidine dimers (CPDs)

Tissue sections (4 μm) were dewaxed and rehydrated using xylol (3 times of 10, 5, and 5 min each) then 100% (3 times of 10, 5, and 5 min each), 95% (5 min), 70% ethanol (5 min), and water (5 min). Then, the antigens were recovered by heat (0.01 M citric acid, pH 6.0) for 10 min at 92°C. Next, the DNA was denatured using 0.2 mM NaOH in 70% ethanol for 30 min. Samples were blocked using 20% fetal bovine serum in 1X PBS for 30 min. Finally, tissues were incubated overnight at 4°C with the primary antibody anti CPD [Clone: TDM2] (1:1500, Cosmo Bio Co. Ltd.) in 5% fetal bovine serum in 1X PBS. After washing with 1X PBS, sections were incubated with anti‐mouse IgG (H + L, 1:200, Vector laboratories) plus DAPI (4′,6‐diamidino‐2‐phenylindole) (1:2000, Invitrogen) in 1X PBS for 1 h at room temperature and then mounted with mounting media (ProLong Gold antifade reagent, Invitrogen). Pictures were taken using the inverted microscope Zeiss Axio Observer 5. CPD intensity normalized to the nuclei area of the epidermis was measured using ImageJ.

### Protein extraction and western blot

A small portion (~2 mm^2^) of the frozen tissue (dorsal skin) collected during the in vivo analysis of SKH‐1 WT and nNOS^+/−^/eNOS^−/−^ mice was first placed into a mortar and submerged in liquid nitrogen until it froze and then rapidly crushed with a pestle and ground into a paste. Next, a mixture of radioimmunoprecipitation assay (RIPA) buffer (100 mM Tris–HCl, 2% v/v Triton X‐100, 300 mM NaCl, 0.2% w/v SDS, 10 mM EDTA, and 1% w/v sodium deoxycholate) with the phosphatase and protease inhibitors, 1X PhosSTOP (Roche, 4906845001) and 1X Complete Mini, EDTA‐free Protease Inhibitor Cocktail (Roche, 11836170001) respectively, was added to the paste and further crushed into a liquid. The liquid was transferred to a smaller glass mortar and pestle over ice to be ground further. The liquid was transferred to a tube and sonicated (Sonic Dismembrator 550, Fisher Scientific, F1996) on power 1.5 for 5 s 3 times. For cell cultures, samples were scraped directly from the plates with RIPA buffer, pipetted, and incubated on ice for 30 min, vortexing them every 5 min. All the lysates were centrifuged at 13,500 *g* at 4°C for 10 min. Protein concentration was measured using the DC protein assay (Bio‐Rad Laboratories, Inc) according to manufacturer instructions. Proteins were separated by SDS‐PAGE. The antibodies GAPDH (1:2000, Santa Cruz Biotechnology, sc‐365062), β‐actin (1:1000, Santa Cruz Biotechnology, sc‐47778), XPC (1:1000, Santa Cruz Biotechnology, sc‐74410), phospho‐p53 (Ser15) (1:1000, Cell Signaling, 9284), p53 (1:1000, Santa Cruz Biotechnology, sc‐126), phospho‐XPA (Ser 196) (1:750, Invitrogen, PA5‐64730), XPA (1:1000, Invitrogen, PA5‐86265), and γ‐H2AX (1:1000, Cell Signaling, 9718) were used.

## Statistical analysis

Statistical significance between tested and control conditions was assessed using Student's *t*‐test (unpaired, two‐tailed), with a *p*‐value ≤0.05 considered statistically significant.

## RESULTS

### cNOS KO mice developed more skin lesions compared with WT mice when exposed to the sUV source

**FIGURE 1:**
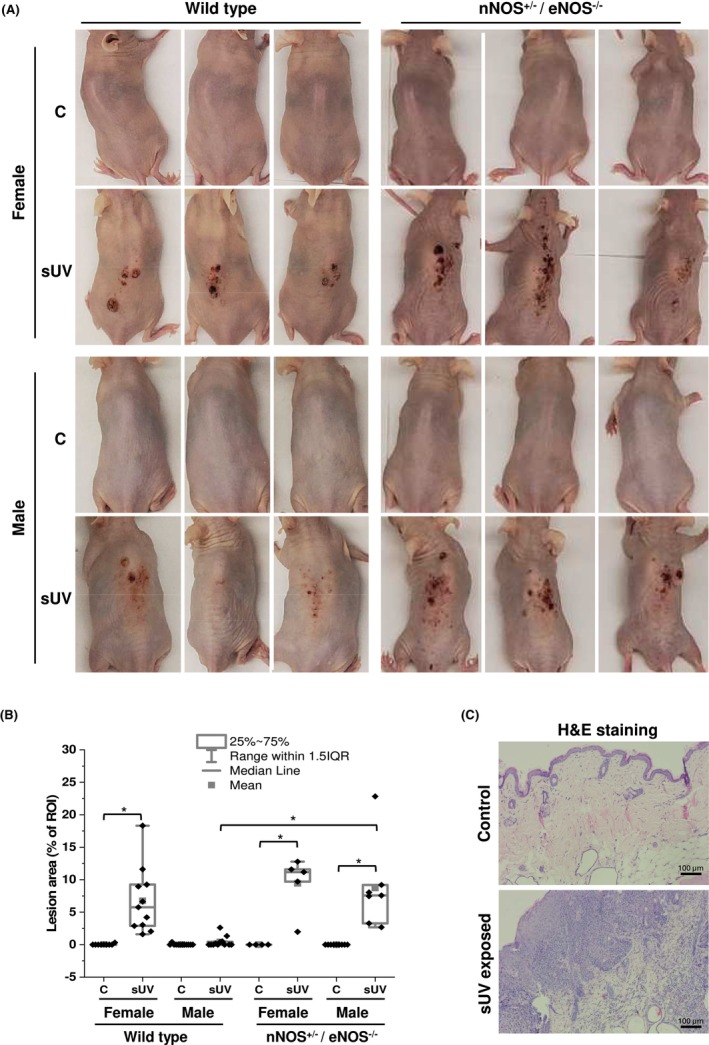
Skin lesion post‐sUV exposure on WT and nNOS^+/−^/eNOS^−/−^ SKH‐1 mice. (A) Images of the skin lesion of 3 representative mice from each group exposed to 180 mJ/cm^2^ of UVB from a solar UV source. Mice were irradiated 3 times a week for 28 weeks. Pictures were taken immediately after euthanasia at week 28. The WT mice exposed to sUV show less severe skin lesions compared with the nNOS^+/−^/eNOS^−/−^ counterparts. (B) Box plot showing the quantification of lesion area relative to a defined region of interest (ROI). The analyzed sample sizes for each group were: Female WT = 9 control and 11 sUV, male WT = 12 control and 12 sUV, female nNOS^+/−^/eNOS^−/−^ = 4 control and 5 sUV, male nNOS^+/−^/eNOS^−/−^ = 9 control and 7 sUV. Boxes represent 25th and 75th percentiles, whiskers indicate 1.5QR, and the line and solid square denote the median and mean, respectively. *p ≤ 0.05. (C) Representative H&E staining of control versus SCC developed in sUV exposed mice. All sUV exposed groups developed lesions consistent with SCC. Bar 100 μm.

Since our previous report demonstrated the role of cNOS in inducing the activation of NF‐κB and the induction of DNA damage post‐UVB in keratinocytes,^6^, ^16^, ^18^ we proceeded to investigate the role of cNOS in sUV‐induced skin cancer formation in a cNOS‐deficient mouse model. We exposed SKH‐1 WT and nNOS^+/−^/eNOS^−/−^ mice to sUV irradiation three times a week for 28 weeks. The results show that in both male and female groups, the WT mice exposed to sUV developed fewer skin lesions compared with the nNOS^+/−^/eNOS^−/−^ mice (Figure 1A). To quantify this observation, the damaged area post‐sUV exposure was quantified and compared between groups. As shown in Figure 1B, lesion areas were significantly larger in the male nNOS^+/−^/eNOS^−/−^ group, while females exhibited a similar trend that did not reach statistical significance. Additionally, analysis of H&E staining in each group of mice exposed to sUV showed that all groups developed squamous cell carcinoma (SCC) (Figure 1C). These results demonstrate that cNOS plays a crucial role in modulating the photocarcinogenic response in mice, although all groups developed SCC following sUV exposure, the cNOS‐deficient mice exhibited a significantly larger lesion area.

### Impaired CPD repair in cNOS knockout cells following solar UV exposure

**FIGURE 2:**
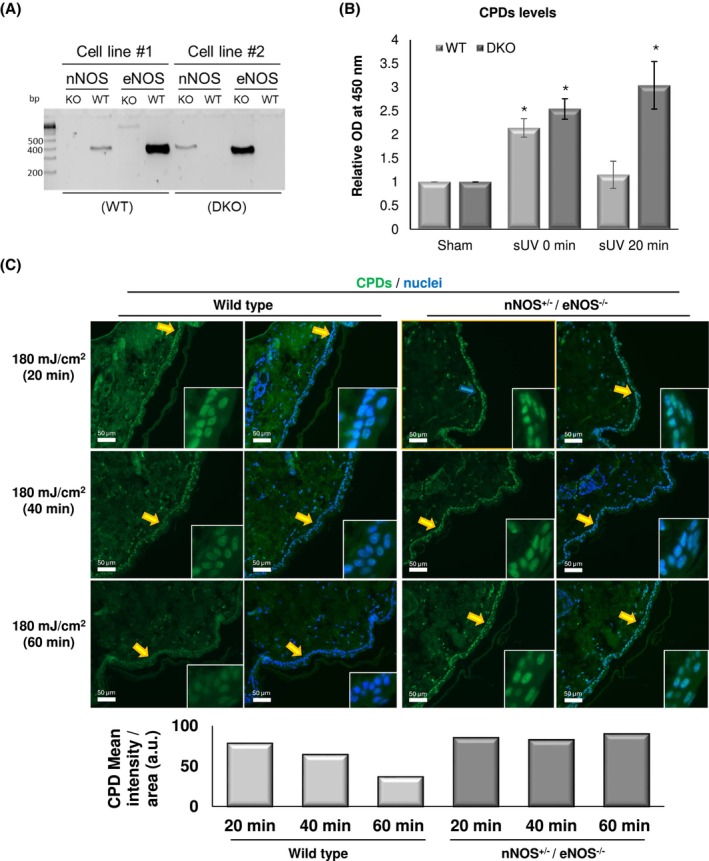
CPDs repair capacity on tissue explants versus primary cells. (A) representative image of the genotyping of the primary fibroblast cells. Samples were run on agarose gel 4%. Cell line # 1 is nNOS and eNOS WT while cell line # 2 is nNOS KO and eNOS KO (DKO). (B) Bar plot showing the quantified levels of CPD detected in primary skin fibroblast isolated from WT and DKO SHK1 mice. The plot shows a quick and significant increase of CPD immediately after an irradiation period of approximately 20 min of sUV and then decreased within 20 min only in the WT cells. *p ≤ 0.05. *N* = 3. Error bars = SD. (C) Pictures show CPD formation in skin explants at 20, 40, and 60 min post‐sUV irradiation. One skin explant per time point collected from a male WT, and a male nNOS^+/−^/eNOS^−/−^ mouse was imaged for each genotype. DAPI (blue) was used as a nuclear counterstain and served as a positional reference to confirm tissue integrity and localization, and the imaging parameters used to acquire the CPD (green) signal were kept constant across all samples and time points. Bar plot displays the mean CPD intensity normalized to the nuclei area of the epidermis for each sample. Yellow arrows indicate the site shown by the insert. Bar 50 μm.

Since DNA damage induced by UV radiation is a main risk factor in skin carcinogenesis, we further explore the involvement of cNOS in CPD formation and repair following sUV exposure. We first examined the formation and early repair dynamics of CPD in primary fibroblast and mouse skin explants. Primary fibroblasts from both WT and nNOS^−/−^/eNOS^−/−^ (DKO) male SKH‐1 mice were isolated (Figure 2A) and either sham‐ or sUV‐irradiated. To assess both CPD formation and the initial repair process, the levels of CPD were measured via ELISA at early points (0 and 20 min) post‐sUV exposure (Figure 2B). The results indicated that CPD levels increased immediately after a relatively long sUV irradiation (approximately 20 min) in both primary cell lines (Figure 2B). However, in WT cells, CPD levels began to decrease 20 min after exposure, suggesting active DNA repair. By contrast, CPD levels in DKO fibroblasts continued to rise during the same time period, indicating impaired repair. This decrease in CPD levels only in the WT fibroblasts post‐sUV exposure suggests that cNOS is playing a role in the repair of CPDs.

To confirm these findings, we evaluated CPD levels and their repair rate using fresh skin explants extracted from male WT and male nNOS^+/−^/eNOS^−/−^ mice.^18^ The results show that CPD levels were observed to decrease from 20 to 60 min post‐sUV exposure only in WT explants, while nNOS^+/−^/eNOS^−/−^ explants exhibited similar CPD levels at the evaluated time points (Figure 2C). The agreement between the results from both the primary fibroblast and the explant model indicates that the expression of the cNOS is needed for the repair of CPDs.

### CPD levels following sUV exposure in HEK293 cells reveal a role for cNOS in DNA repair

**FIGURE 3:**
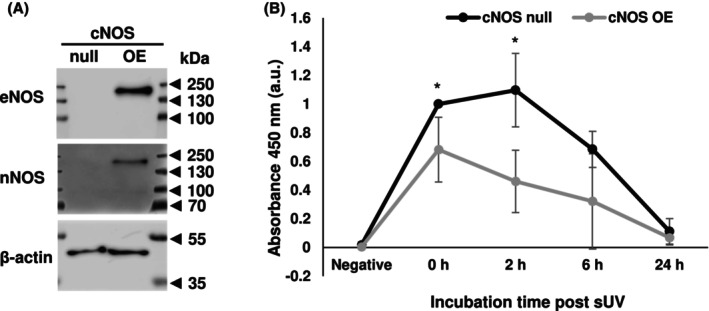
CPD levels following sUV exposure in HEK293 cells reveal a role for cNOS in DNA repair. (A) western blot confirming the establishment of HEK293 cells stably expressing both cNOS (nNOS and eNOS). (B) Plot showing the levels of CPD measured via ELISA on samples collected from HEK293 cNOS‐null and cells stably expressing cNOS (cNOS OE) at 0, 2, 6, and 24 h post‐sUV exposure (180 mJ/cm^2^ and irradiation period of ~20 min). *p ≤ 0.05 (cNOS null vs. OE). *N* = 4. Error bars = SD.

To explore the role of cNOS in UV‐induced CPD repair, HEK293 cells, which are cNOS null, were used to create stable cNOS‐expressing cell lines (cNOS OE). The successful expression of both cNOS isoforms (eNOS and nNOS) in these cells was confirmed by western blot analysis (Figure 3A). CPD levels were assessed via ELISA at 0, 2, 6, and 24 h post‐sUV exposure. The data demonstrated that CPD levels at time 0 h and 2 h were significantly lower in the cNOS OE cells than the levels at the same time points observed in the HEK293 cNOS‐null cells (Figure 3B). Moreover, only the cNOS OE cells showed decreased levels of CPD at 2 h post‐sUV exposure compared to the 0 h, which indicated the repair of this type of DNA damage started early after UV exposure. By contrast, the HEK293 cNOS‐null cells not only showed higher levels of CPD at 0 h after sUV exposure but also an initial slow removal of them. After the 2 h time point, the slope of the curve for cNOS OE cells suggests a deceleration in CPD removal compared with cNOS‐null cells. However, both cell lines were able to show a complete removal of CPD at 24 h post‐sUV. These results indicate a role for cNOS in the early phase of CPD's repair.

### Evaluation of the NER pathway in cNOS‐null and cNOS overexpression (OE) HEK293 cells

**FIGURE 4:**
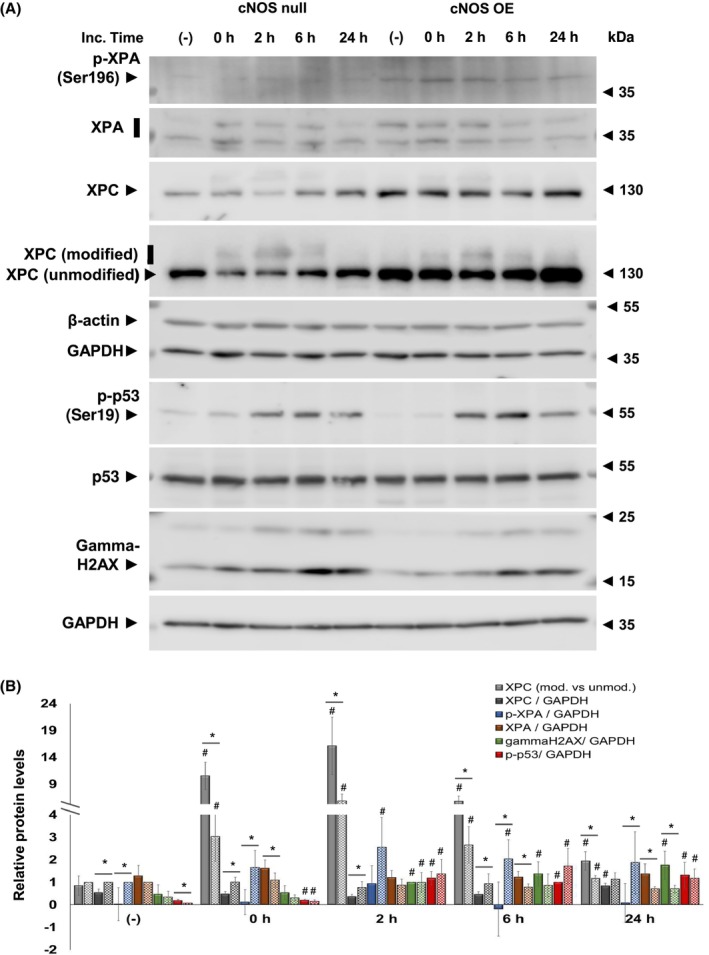
Cells lacking cNOS might have higher accumulation of damaged DNA. (A) Representative western blots showing the proteins levels of XPA, XPC, gamma‐H2AX, and p53 in HEK293 cNOS‐null or cells overexpressing cNOS (cNOS OE) measured at 0, 2, 6, and 24 h post‐sUV exposure. *N* ≥ 3. (B) Bar plot showing the quantification of the proteins shown in panel A (modified vs. unmodified XPC = light gray; XPC = dark gray; p‐XPA = blue; total XPA = brown; gamma‐H2AX = green; p‐p53 = red). *p ≤ 0.05 cNOS null (solid fill) versus cNOS OE (stripes fill). ^#^ *p* ≤ 0.05 irradiated versus sham (−) samples. *N* ≥ 3. Error bars = SD.

To investigate if cNOS affects the NER pathway, we analyzed the total levels of XPC and the levels of phosphorylated XPA in cNOS‐null and cNOS OE HEK293 cells. Both XPC and XPA play important roles in the recognition and removal of CPDs via NER.^21^, ^22^, ^23^, ^24^, ^25^ Our data showed that the basal levels of XPC were significantly higher in cNOS OE cells except at 24 h (Figure 4A,B). In both cNOS‐null and cNOS OE cells, XPC protein levels slightly decrease and then increase post‐sUV. However, the increase was only significant at 24 h after sUV exposure in the cNOS‐null cells (Figure 4A,B). In addition, a band of higher molecular weight, which could correspond to post‐translationally modified XPC,^26^, ^27^, ^28^ is observed at 0, 2, and 6 h post‐sUV (Figure 4A,B). The levels of this post‐translational modified XPC versus unmodified XPC were significantly higher in the cNOS‐null cells at the 0, 2, 6, and 24 h time points (Figure 4A,B). Additionally, the levels of phosphorylated XPA were also significantly higher in cNOS OE cells and increased significantly post‐sUV irradiation at 2 and 6 h (Figure 4A,B). Total XPA levels were significantly lower at 0, 6, and 24 h in the cNOS OE cells than that in the cNOS‐null cells. Moreover, total XPA decreased significantly in the cNOS OE cells at 24 h post‐sUV (Figure 4A,B).

To better understand the role of cNOS on CPD's repair, we also evaluated the levels of p53 phosphorylated in Ser15 and the levels of gamma‐H2AX in cNOS‐null and cNOS OE HEK293 cells. The phosphorylation of p53 at Ser15 and of Ser139 of the histone variant H2AX are known to occur in response to UV radiation and DNA damage.^29^, ^30^, ^31^, ^32^, ^33^, ^34^ Increased levels of phosphorylated p53 in both cell lines from 2 h post‐sUV exposure were detected (Figure 4A,B). By contrast, the levels of gamma‐H2AX are observed to increase significantly post‐sUV in both cell lines, showing higher protein levels in the cNOS‐null cells at 24 h post‐sUV (Figure 4A,B). Additionally, the results indicate the levels of gamma‐H2AX decreased faster in the cNOS OE cells compared with the cNOS‐null cells (Figure 4A,B). Both phosphorylated p53 and gamma‐H2AX stay elevated even at 24 h post‐sUV (Figure 4A,B). This result suggests that cells lacking cNOS might have more DNA damage or a prolonged DNA damage response.

### Determination of XPC and XPA expression in cNOS‐deficient mice post‐sUV exposure

**FIGURE 5:**
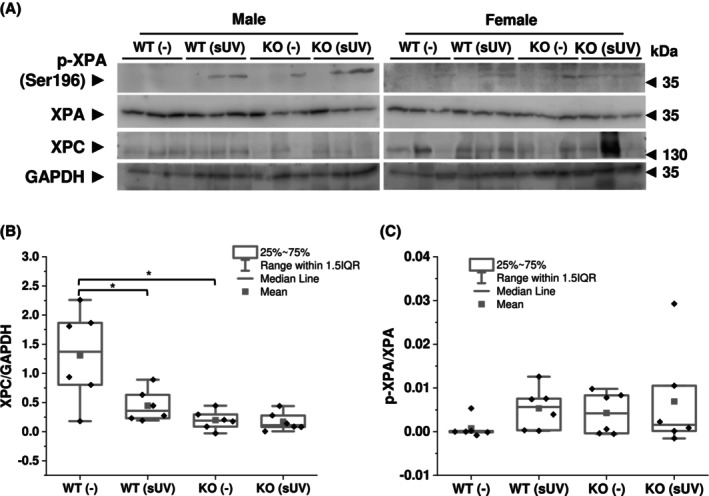
XPA and XPC protein levels on WT and nNOS^+/−^/eNOS^−/−^ SKH‐1 mice. Western blot analysis and box plot showing the levels of XPC (A, B) and phosphorylated XPA (A, C) on tissue samples from male and female WT and nNOS^+/−^/eNOS^−/−^ SKH‐1 mice. *N* = 6 mice per group (3 males and 3 females). Boxes represent 25th and 75th quartiles, whiskers represent 1.5QR, line and solid square represent median line and mean, respectively. *p ≤ 0.05.

We also evaluated the levels of XPC and XPA in tissue collected from three male and three female WT and nNOS^+/−^/eNOS^−/−^ SKH‐1 mice per condition, following 28 weeks of irradiation. However, a big dispersion in the protein levels between each group was observed (Figure 5). For XPC, we observed that WT mice expressing cNOS exhibited statistically significant higher basal levels of XPC protein compared with cNOS‐deficient mice (Figure 5A,B). Notably, sUV exposure led to a significant decrease in XPC levels only in WT mice (Figure 5A,B). By contrast, for XPA, we observed a trend toward increased phosphorylation of XPA in WT mice after sUV exposure (*p* = 0.06), whereas this increase was not evident in cNOS‐deficient mice (*p* = 0.61) (Figure 5A,C). These results demonstrate that after sUV exposure, the lack of cNOS affects the expression levels of the NER proteins XPC and XPA, which might be reducing or affecting the proper activation of the NER pathway.

## DISCUSSION

CPD formation is a signature of ultraviolet radiation‐induced damage and plays a significant role in skin carcinogenesis. Traditionally, NO^˙^ and cNOS functions have been associated with CPD formation and have been reported to restrict DNA repair responses.^11^, ^35^ However, in this report, we demonstrate that the proper regulation of cNOS expression is crucial for an effective response to DNA damage induced by sUV exposure, particularly in the repair of CPD. Our findings suggest that, in addition to its known roles in genotoxic stress, basal levels of cNOS‐derived NO^˙^ may also play a protective role by promoting CPD repair. This introduces a more nuanced understanding of the role of NO^˙^ in photobiology, suggesting that it functions not only as a potential DNA‐damaging agent but also as a regulator of the DNA damage response when maintained at physiological levels.

Our data from in vivo experiment (Figure 1) reveals that cNOS‐deficient mice develop more skin lesions compared with WT mice following chronic sUV exposure. While this may suggest a role for impaired DNA repair, it is important to acknowledge that lesion severity could also result from multiple systemic factors influenced by cNOS deficiency, including alterations in immune response, skin physiology, oxidative stress, and vascular function. Therefore, further studies are required to distinguish the direct effects of cNOS on DNA repair from these secondary physiological consequences. While excessive NO^˙^ is known to contribute to oxidative stress, cellular damage, and hinder DNA repair mechanisms,^35^, ^36^, ^37^ our findings strongly suggest that basal levels of nitric oxide are also essential for modulating repair processes following UV‐induced damage. To further investigate the role of cNOS in DNA repair, we examined CPD repair in fibroblasts and skin explants derived from the same SKH‐1 mouse model. The experiments shown in Figure 2 clearly demonstrate that cNOS deficiency results in delayed CPD removal, supporting the notion that cNOS activity facilitates timely DNA repair. These findings not only complement our in vivo observations but also provide a mechanistic explanation for the increased skin lesion formation observed in cNOS‐deficient mice. Importantly, by utilizing both primary fibroblasts and tissue explants derived from the same animals, we minimize technical variability and strengthen the biological relevance of our results. Interestingly, primary fibroblasts isolated from the same SKH‐1 mice exhibited relatively rapid CPD repair under our experimental conditions, an observation that is uncommon in the literature and may reflect active DNA damage removal mechanisms in our system. The rapid removal of CPDs observed in our primary fibroblast cultures may reflect intrinsic characteristics of this specific cell line. Factors such as transcriptional activity, chromatin accessibility, or DNA repair enzymes expression could accelerate lesion removal. Since this rapid repair was not observed in HEK293 (Figure 3B) or HaCaT cells exposed under identical conditions, we believe this is a cell‐type‐specific response rather than an artifact of the UV source or dose. Nonetheless, we cannot entirely exclude the possibility that the CPDs generated by the UVA‐340 lamp differ in structure or distribution compared to those induced by conventional UVB sources. This lamp emits a relatively low percentage of short‐wavelength UVB and a higher proportion of longer‐wavelength UVA, which may influence lesion formation and repair dynamics.^16^

Additionally, by utilizing HEK293 cells, which are cNOS‐null, we investigated the kinetics of CPD removal at longer time and the underlying mechanisms contributing to the observed protective response. Since the data obtained from primary fibroblasts were limited to early time points, the extended analysis using HEK293 cells provided a more comprehensive view of CPD removal dynamics. These results support the hypothesis that cNOS expression correlates with reduced early CPD levels, possibly due to either reduced lesion formation or enhanced initial repair (given that the irradiation period is approximately 20 min, it is possible that some CPD repair occurs during exposure). Notably, a decrease in CPD levels at the 2‐h mark was only observed in cNOS OE cells (Figure 3B). This finding suggests a potential role for cNOS in promoting rapid initial repair. However, examination of the slope of the repair curves after the 2‐h point indicates that the repair rate in cNOS OE cells becomes slower compared with cNOS‐null cells, suggesting a biphasic effect of cNOS on CPD repair kinetics. These results imply that cNOS may modulate the kinetics of CPD removal, although further studies will be required to fully elucidate its mechanistic role. Our results also showed that the genetic depletion of cNOS led to increased levels of gamma‐H2AX (Figure 4), which can reflect an intensified DNA damage response and the accumulation of DNA repair intermediates due to impaired CPD removal.^33^, ^38^ This is further supported by the lower levels of XPC and phosphorylated XPA in the cNOS‐null cells (Figure 4), which correlate with higher initial CPD levels and slower removal following UV exposure, hallmarks of inefficient DNA repair.^26^, ^39^, ^40^ These findings suggest that cNOS activity may influence NER not only by regulating the expression levels of key repair proteins such as XPC and XPA, but also by potentially affecting their post‐translational modifications. In particular, cNOS may modulate XPC ubiquitination or SUMOylation, which are important for its protein stability, DNA‐binding capacity, and repair efficiency.^28^, ^41^, ^42^ Overall, the HEK293 cell model reinforces our observations and provides mechanistic insight into the role of cNOS in supporting effective CPD repair following sUV exposure. However, since gamma‐H2AX is a general marker of DNA damage and can also reflect checkpoint activation or other types of lesions, we recognize its limitations in specifically indicating unrepaired CPDs. We therefore focused on direct CPD quantification as a more lesion‐specific measure. We also acknowledge that no direct NER activity assays were performed in this study, and thus, the effect of cNOS on overall repair kinetics remains to be confirmed.

Although uncommon, other examples of the cytoprotective effects of nitric oxide generation can be found in the literature.^43^, ^44^ For instance, fibroblasts transfected with inducible NOS (iNOS) exhibit a modest reduction in single‐strand break susceptibility following hydrogen peroxide treatment,^43^ suggesting a protective role under specific conditions. While the mechanisms underlying these observations remain unclear, our data suggest that the absence of basal cNOS expression in cNOS‐deficient models may impair or delay the activation of DDR signaling pathways. Although we did not directly measure NO^˙^ levels in the current study, our previous work has shown that pharmacological inhibition of cNOS significantly reduces UV‐induced NO^˙^ production under similar conditions.^7^, ^45^, ^46^ This supports the use of cNOS expression as a proxy for NO^˙^ activity in our experimental models. This is consistent with the lower expression of XPC and phosphorylated XPA observed in cNOS‐deficient cells and mice, compared with their cNOS‐expressing counterparts (Figures 4 and 5). It is possible that constitutive NO^˙^ production acts not merely as a reactive species but also as a redox‐sensitive signaling mediator, facilitating early DNA repair protein recruitment or transcriptional activation. Additionally, cNOS may influence mitochondrial function or apoptotic signaling, as suggested by our preliminary data showing increased apoptosis and reduced viability in cNOS‐overexpressing HEK293 cells upon UVB irradiation (unpublished). While this may initially appear detrimental, increased apoptosis could reflect enhanced clearance of UV‐damaged cells, thereby reducing the risk of malignant transformation. The precise mechanisms through which cNOS modulates UV‐induced DNA damage responses and contributes to protection against skin carcinogenesis remain to be fully elucidated, particularly given the complexity of cellular responses to UV stress. Factors such as oxidative stress, cellular senescence, inflammatory signaling, or aging‐related shifts in NO^˙^ sensitivity may all modulate this outcome.^47^, ^48^

**FIGURE 6:**
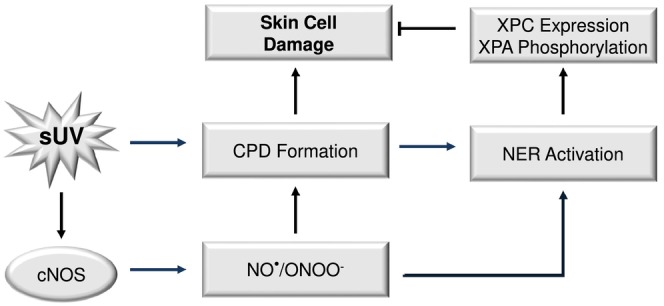
Proposed model for the dual role of cNOS in DNA damage and repair upon UV radiation.

In conclusion, and as summarized in Figure 6, our study introduces a new perspective on the role of NO^˙^ and cNOS in skin carcinogenesis: cNOS is not only involved in modulating basal NO^˙^ levels but also essential for protecting cells from DNA damage and enabling proper activation of the NER pathway following sUV exposure (Figure 6). These findings challenge the prevailing notion that NO^˙^ is solely deleterious in the context of sUV‐induced stress and highlight its dualistic nature as both a damaging and protective factor, depending on its source and concentration. By demonstrating that cNOS activity supports DNA repair efficiency and limits sUV‐induced skin damage, the study has implications for understanding skin aging, photoprotection, and cancer susceptibility. Preserving or enhancing physiological NO^˙^ signaling may represent a novel strategy for mitigating sUV‐induced genotoxicity and maintaining genomic integrity in skin cells.

## Data Availability

The data that support the findings of this study are available from the corresponding author upon reasonable request.
